# Combined DFT and Kinetic Monte Carlo Study of UiO-66 Catalysts for γ-Valerolactone Production

**DOI:** 10.1021/acs.jpcc.3c06053

**Published:** 2024-01-12

**Authors:** Thanh-Hiep Thi Le, David Ferro-Costas, Antonio Fernández-Ramos, Manuel A. Ortuño

**Affiliations:** † Centro Singular de Investigación en Química Biolóxica e Materiais Moleculares (CIQUS), Universidade de Santiago de Compostela, 15782 Santiago de Compostela, Spain; ‡ Departamento de Química Física, Facultade de Química, Universidade de Santiago de Compostela, 15782 Santiago de Compostela, Spain

## Abstract

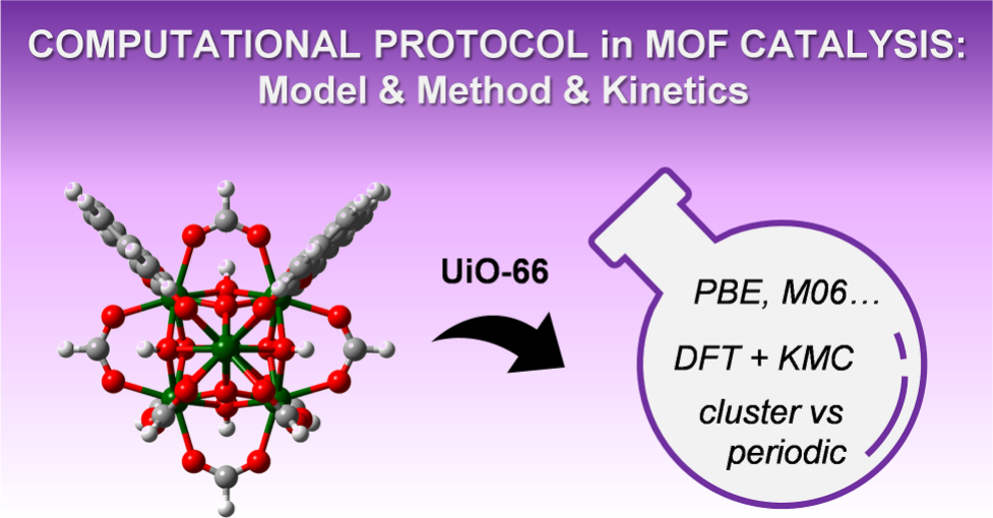

Zr-based metal–organic frameworks (MOFs) are excellent heterogeneous porous catalysts due to their thermal stability. Their tunability via node and linker modifications makes them amenable for theoretical studies on catalyst design. However, detailed benchmarks on MOF-based reaction mechanisms combined with kinetics analysis are still scarce. Thus, we here evaluate different computational models and density functional theory (DFT) methods followed by kinetic Monte Carlo studies for a case reaction relevant in biomass upgrading, i.e., the conversion of methyl levulinate to γ-valerolactone catalyzed by UiO-66. We show the impact of cluster versus periodic models, the importance of the DF of choice, and the direct comparison to experimental data via simulated kinetics data. Overall, we found that Perdew–Burke–Ernzerhof (PBE), a widely employed method in plane-wave periodic calculations, greatly overestimates reaction rates, while M06 with cluster models better fits the available experimental data and is recommended whenever possible.

## Introduction

Metal–organic frameworks (MOFs) have emerged as highly promising materials with significant potential for chemical catalysis. MOFs consist of metal ions or clusters (i.e., nodes) connected by organic ligands (i.e., linkers) which form porous crystalline frameworks with an expansive surface area and tunable pore size.^1^ One of the most intriguing attributes of MOFs as catalysts is their ability to exert precise control over catalytic activity and selectivity.^2,3^ The porous structure of MOFs provides a confined and regulated environment for the formation of unique active sites, which can augment the reaction rates and selectivity of various chemical reactions. This is particularly true for MOFs with Zr-based nodes, which are highly stable and amenable for catalyst design.^4^

**Figure 1:**
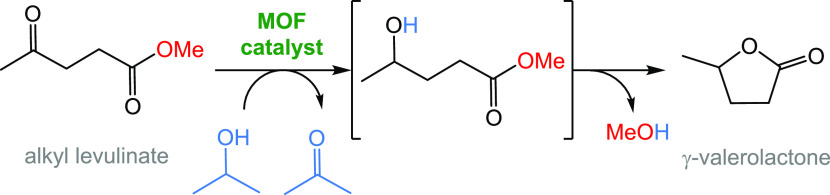
MOF-catalyzed conversion of methyl levulinate to γ-valerolactone via catalytic hydrogen transfer.

One recent application of MOFs is the catalytic upgrade of biomass-based substrates. The conversion of biomass into valuable chemicals and fuels presents an alluring alternative to conventional fossil fuel-based processes, offering a more sustainable approach.^5,6^ Of particular interest is the production of the platform chemical γ-valerolactone (GVL)^7^ from levulinic acid and levulinate esters (e.g., methyl levulinate or ML) via catalytic transfer hydrogenation, i.e., using alcohols as hydrogen source (Figure 1).

Researchers have reported the use of MOF catalysts for this reaction, such as UiO-66,^8−10^ MOF-808,^8,11^ ZrF,^12^ and DUT-52.^13^ However, from a rational design perspective, a detailed understanding of the operating mechanisms involved in the MOF-catalyzed processes is indeed needed.

To address this issue, we will rely on computational modeling via density functional theory (DFT) to investigate MOF-catalyzed processes at an atomic level of detail.^14−16^ Regarding materials modeling, one has two main approaches: periodic or cluster. On the one hand, extended periodic models describe the crystalline structure of the material; they are ideal for describing confinement effects but are mostly constrained to density functionals (DFs) of the generalized gradient approximation (GGA) family. On the other hand, finite-size cluster models represent only a section of the full material; they are appropriate for studying active sites in a local environment and a wide range of DF methods (and even multiconfigurational^17^) are available at reasonable computational costs. Following those methodologies, several computational studies have been reported concerning biomass-related processes.^18^ Related to our reaction of interest, the catalytic transfer hydrogenation of furfural to furfuryl alcohol has been performed with cluster UiO-66^19^ and cluster MOF-808.^20^ And very recently, the conversion of methyl levulinate to γ-valerolactone has been studied with periodic UiO-66^21^ and cluster MOF-525.^22^

The proposed reaction mechanisms seem feasible under the reported conditions, but the lack of detailed kinetic studies hampered a direct comparison with experiments. In other words, DFT alone is usually not enough to confirm reaction mechanisms and kinetics analyses are needed. These simulations are thus typically coupled with microkinetic modeling (MKM).^23^ Regarding the production of GVL, such a combined DFT-MKM approach has been carried out for Ru(0001) surface catalysts.^24,25^ As for Zr-based MOFs, there exist DFT-MKM studies for UiO-67,^26^ NU-1000,^27^ and MOF-808.^28^ While MKM is clearly a powerful tool, it may present some limitations such as lower accuracy, sensitivity to initial conditions, and limited transferability to different reaction systems.^29^ Even in some difficult cases, biasing DFT-computed Gibbs energies is necessary to properly fit the experimental data.^30^ Alternatively, DFT can also be combined with kinetic Monte Carlo (KMC), which can surpass mean-field MKM and make it a truly first-principles approach.^29,31^ This strategy holds promise for modeling biomass conversion processes, offering a comprehensive understanding of energetics and kinetics involved; however, the literature on MOF-based catalysis is still scarce.^32^

Herein, we perform a computational study of the conversion of methyl levulinate into γ-valerolactone with isopropanol as a hydrogen donor source, as reported experimentally.^8−10^ We employ a cluster model of UiO-66, which enables a more diverse description of reaction energies, transition states, and barrier heights in terms of DFs, such as GGA, meta-GGA, and hybrid methods. Finally, we carried out KMC simulations to predict the rate-determining steps and reaction rates, allowing us to assess the feasibility and accuracy of the computational protocol against experimental data.

## Methods

### Computational Models

**Figure 2:**
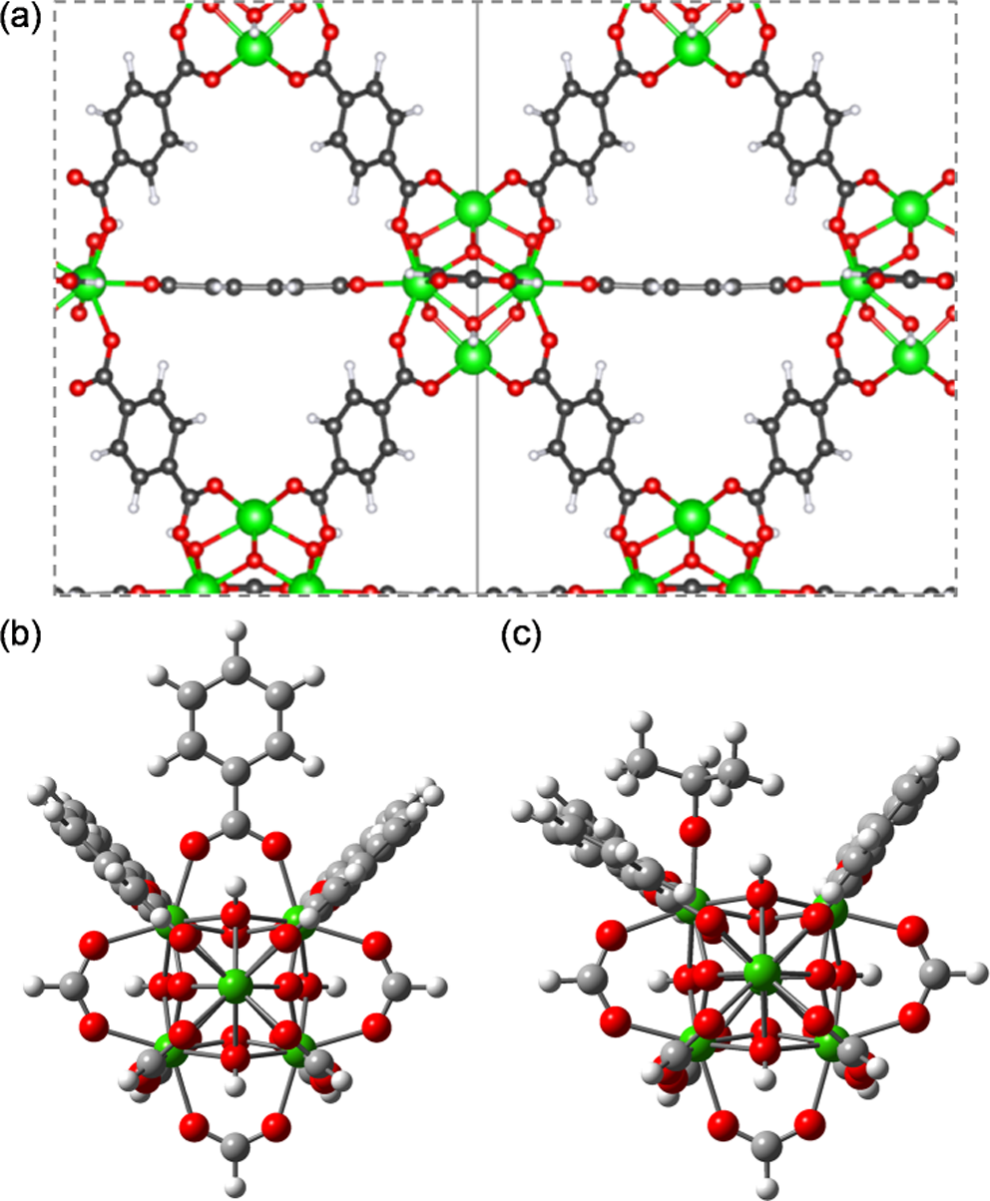
Structure of UiO-66: (a) periodic model, (b) pristine cluster model, and (c) defective ^*i*^PrO-bound cluster model. Atom legend: Zr = green, O = red, C = gray, and H = white.

The UiO-66 MOF consists of [Zr_6_O_4_(OH)_4_] nodes and 1,4-benzenedicarboxylate linkers (Figure 2a).^33^ A cluster model was derived from previous DFT-optimized periodic calculations.^21,34^ The cluster has one [Zr_6_O_4_(OH)_4_] node connected to 12 linkers: five benzoate groups near the active site and seven formate groups (Figure 2b). The *para*-carbon atoms of the benzoate groups were kept fixed to simulate the rigidity of the framework. It is known that missing linkers create defective nodes,^35^ where Zr atoms are capped by OH and H_2_O.^36,37^ However, since our experimental system takes place in isopropanol solution,^10^ we assume that OH/H_2_O are eventually replaced by ^*i*^PrO/^*i*^PrOH. Thus, we create a model where one benzoate is exchanged by isopropoxide, maintaining charge neutrality (Figure 2c).

### Density Functional Theory

DFT calculations were carried out with Perdew–Burke–Ernzerhof (PBE)^38,39^ and Grimme D3 dispersion scheme^40^ as implemented in Gaussian 16.^41^ Zr atoms were described using the Stuttgart effective core potentials (SDD)^42^ and def2-TZVP; O, C, and H atoms were described using def2-SVP.^43,44^ Geometry optimizations were performed in the gas phase. All conformations of ML were considered and computed using the Torsiflex program.^45^ Transition states were verified to connect with the corresponding reactants and products by following the normal modes associated with their imaginary frequencies. All frequencies below 50 cm^–1^ were shifted to 50 cm^–1^ when computing vibrational partition functions at 403.15 K.^46^ After geometry optimization, single-point calculations were performed using def2-TZVP/SDD for Zr and def2-TZVP for O, C, and H.^43,44,47^ The same approach was followed for the DFs PBE0-D3,^40,48^ M06-L,^49^ and M06.^50^

All energies and geometries reported herein are available in the open-access^51^ ioChem-BD platform^52^ through the following database.^53^

### Reaction Dynamics Calculations

The reaction network for the conversion of ML to GVL consists of several elementary reaction steps. The rate constants were initially calculated by conventional transition state theory (TST)^54^ or by multistructural transition state theory (MS-TST)^55^ in the case of multiple torsional conformers. These calculations were complemented by canonical variational transition state theory (CVT)^56^ when suspecting that variational effects could play a role. The variational coefficient for a given reaction is just the ratio between the rate constant evaluated at the maximum of the Gibbs energy along the minimum energy path (MEP) and the rate constant considering that the bottleneck for the reaction is at the transition state. The MEP was followed employing the Page-McIver algorithm^57^ with a gradient step size of 0.01 Bohr and Hessian calculations every 9 steps.

The global mechanism was simulated by kinetic Monte Carlo (KMC) employing as input data the experimental conditions. KMC is a stochastic method that provides the evolution of the population (concentration) of species with time. Details about the KMC method can be found elsewhere.^58,59^ All dynamics calculations were performed with a modified version of the Pilgrim software.^59^

## Results and Discussion

The outline of the article is as follows. First, we compute the reaction mechanism at the DFT level and evaluate the impact of cluster and periodic models. We then assess the influence of the computational method by reoptimizing intermediates and transition states with different DFs. Finally, we employ KMC to benchmark the previous calculations against experimental data.

### Periodic vs Cluster Models

As mentioned previously, we prepared a defective finite-size cluster from the periodic structure of UiO-66 (Figure 2) to study the reaction mechanism for the conversion of ML to GVL.

**Figure 3:**
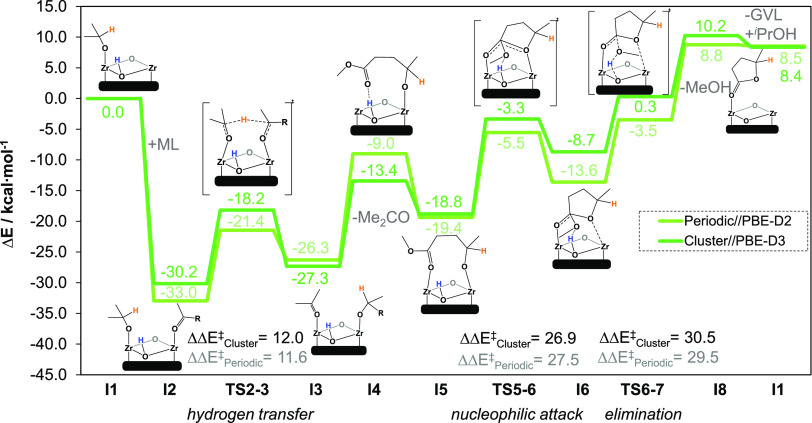
Electronic energy profiles (in kcal·mol^–1^) of defective UiO-66 using cluster models at PBE-D3 level and periodic models at PBE-D2 level.^21^ R = (CH_2_)_2_CO_2_Me.

Figure 3 shows the electronic energy profile obtained for both periodic (previous work^21^) and cluster (this work) models. Since the mechanism is shared for both models, from now on only cluster electronic energies are discussed. Initially, the catalytic active site in **I1** contains an ^*i*^PrO group and one free Zr site for ML to bind via the carbonyl group, forming **I2** (−30.2 kcal·mol^–1^). Subsequently, hydrogen transfer from ^*i*^PrO to activated-ML occurs via **TS2–3** (−18.2 kcal·mol^–1^), with an energy barrier of 12.0 kcal·mol^–1^. This step leads to the formation of **I3** (−27.3 kcal·mol^–1^) and the release of acetone to generate **I4** (−13.4 kcal·mol^–1^). From there, the substrate undergoes a conformational change to form more stable bidentate species **I5** (−18.8 kcal·mol^–1^). Next, a nucleophilic attack takes place via **TS5–6** (−3.3 kcal·mol^–1^) with an energy barrier of 26.9 kcal·mol^–1^. After the formation of **I6** (−8.7 kcal·mol^–1^), the reaction continues with the elimination of MeOH, which is assisted by the μ_3_–OH group of the node^60,62^ via **TS6–7** (0.3 kcal·mol^–1^) with an energy barrier of 30.5 kcal·mol^–1^. In **I8**, MeOH is released and GVL is already formed and bound to Zr (10.2 kcal·mol^–1^). Finally, GVL releases and one molecule of ^*i*^PrOH regenerates the catalytic species **I1** (8.4 kcal·mol^–1^).

When comparing periodic and cluster models,^60^ the geometries of intermediates and transition states are essentially the same. The electronic energy profiles and their corresponding activation energies are very similar for both (Figure 3). The major energy differences (ca. 4 kcal·mol^–1^) arise in **I4**, **I6**, and **TS6–7**. However, the relative activation barriers remain the same, within 1 kcal·mol^–1^; thus, it is reasonable to assume that both models perform similarly. The different dispersion schemes (Figure S1) and diffuse function basis sets (Figure S2) do not affect that outcome.

### Extended Mechanism

Since the previous mechanism at the periodic DFT level was missing some steps, we have added new intermediates and transition states to the reaction profile. In particular, we have added one explicit ^*i*^PrOH molecule^61^ and computed the catalyst regeneration. The extended mechanism involves the elementary reactions listed below:
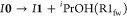

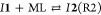

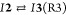

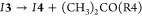

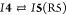

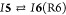

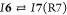

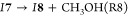

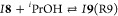

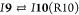

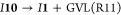

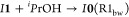
Reactions R1–R11 can be classified into four main steps: hydrogen transfer, nucleophilic attack, elimination, and regeneration.

**Figure 4:**
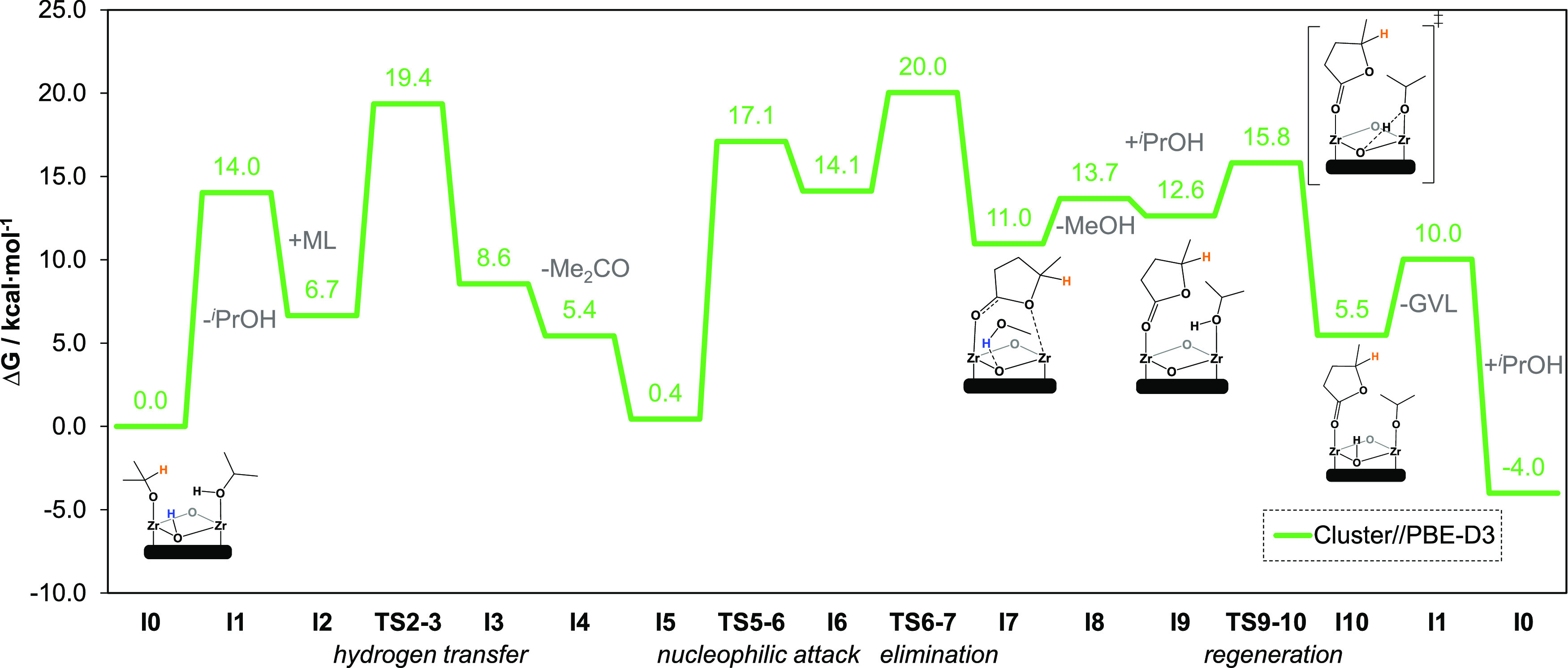
Gibbs energy profile of the extended mechanism at 403.15 K (in kcal·mol^–1^) of defective UiO-66 using cluster models at the PBE-D3 level.

For a proper future comparison with experimental data, we report Gibbs (free) energies from now on. The Gibbs energy profile at the PBE-D3 level is shown in Figure 4. The new intermediate **I0** is set to zero energies and is now isoenergetic with **I5**. The rest of the mechanism is the same as before except for the additional last step, the catalyst regeneration. There, we report the dissociative substitution of MeOH in **I7** by ^*i*^PrOH in **I9** and the reprotonation of the node via **TS9–10** to yield the initial active species. The most demanding steps are the hydrogen transfer with 19.4 kcal·mol^–1^ and the elimination with 20.0 kcal·mol^–1^.

To investigate the additional factors that may affect the energy barriers, we next computed the reaction profiles of the extended reaction mechanism with other density functionals.

### Impact of the Density Functional

While plane-wave-based periodic simulations are mostly confined to GGA DFs due to computing time, the use of cluster models allows for the evaluation of a wide range of DFs. This is particularly important in catalytic processes,^63^ where GGA DFs such as PBE are known to underestimate activation barriers,^64,65^ including zeolite chemistry^66,67^ and ZrO_2_ clusters.^68^

**Figure 5:**
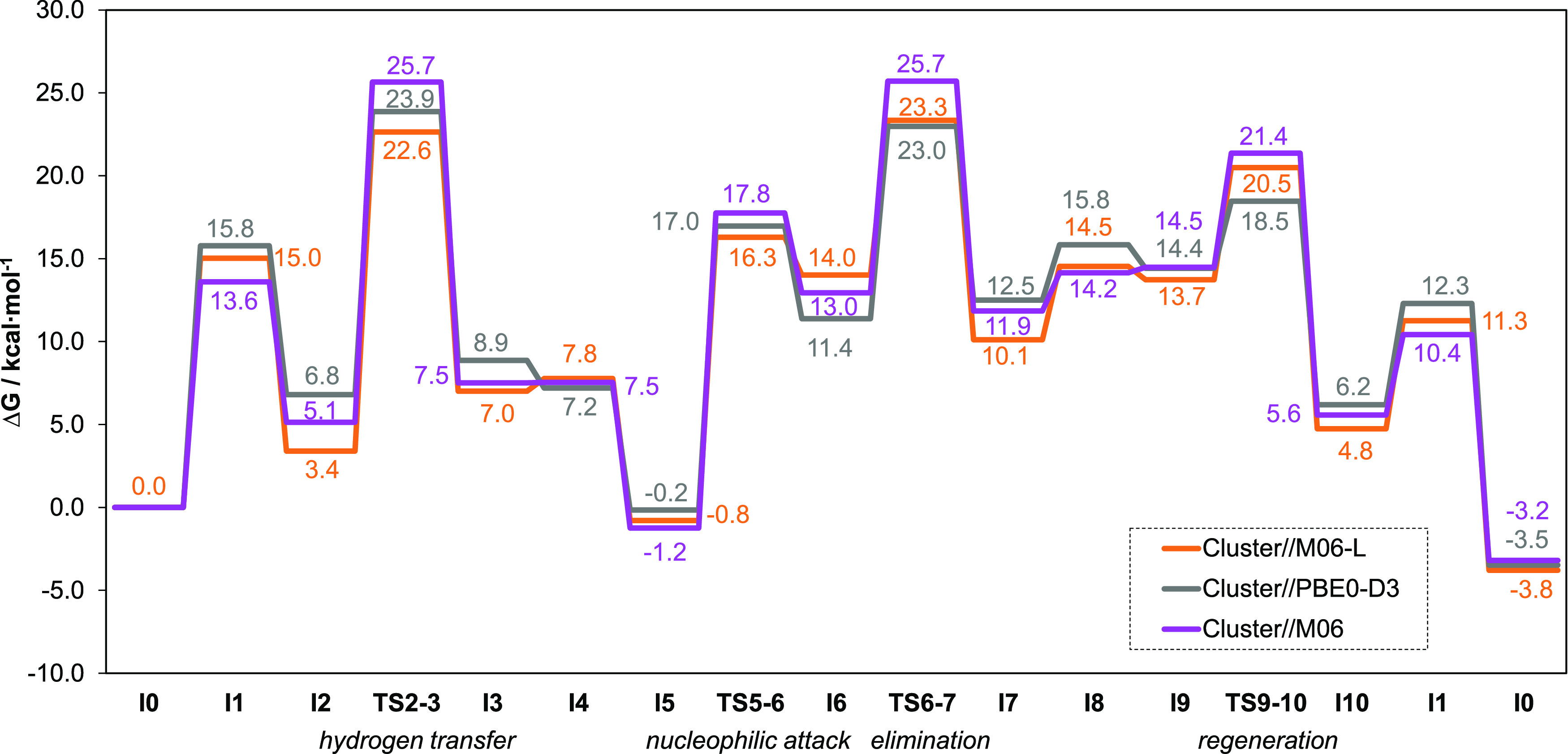
Gibbs energy profiles of the extended mechanism at 403.15 K (in kcal·mol^–1^) of defective UiO-66 using cluster models at the M06-L, PBE0-D3, and M06 levels.

To evaluate the impact of the DF of choice in our system, we recomputed the previous PBE-D3 mechanism with three more DFs: hybrid PBE0-D3 (25% exact exchange) for comparison with its GGA counterpart; meta-GGA M06-L for its extensive use in Zr-based nodes;^69^ and hybrid M06 (27% exact exchange) for its general applicability to transition metals.^70^ The Gibbs energy profiles for the conversion of ML into GVL with the three DFs are shown in Figure 5. We observe similar optimized geometries and transition states across all of the DFs employed (Table S3). Energy differences between DFs in intermediates and transition states are ca. 1–4 and 3–7 kcal mol^–1^, respectively.

**Figure 6:**
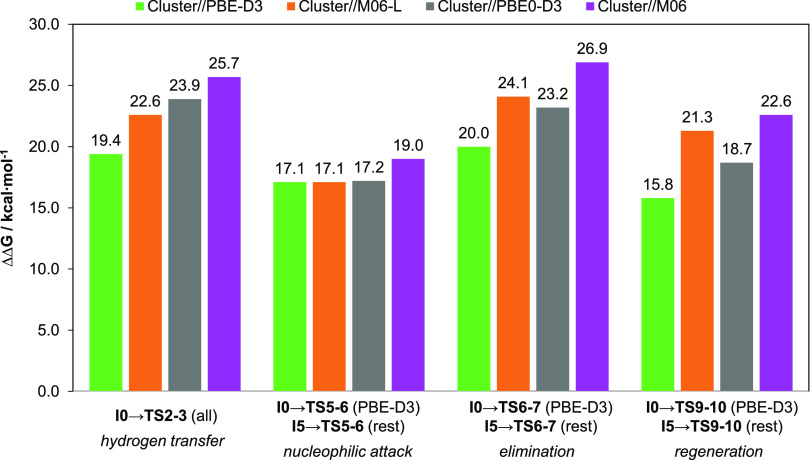
Gibbs energy barriers (in kcal·mol^–1^) for the mechanistic steps at the PBE-D3, M06-L, PBE0-D3, and M06 levels. All barriers are computed using the TS and the prior most stable intermediate.

The Gibbs energy barriers for all four DFs are summarized in Figure 6. Looking at the type of chemical transformation, we observe that the intramolecular nucleophilic attack process does not change, but the rest are sensitive to the DF. It is worth noting that those steps present a hydrogen shift,^71,72^ from the ^*i*^PrO group in **TS2–3** and from/to the μ_3_–OH of the node in **TS6–7/TS9–10**. As for DFs, we notice that PBE-D3 provides the lowest energy barriers. This is followed by PBE0-D3, M06-L, and M06, which give the highest ones.^62^ In all cases, the hydrogen transfer and elimination process seem to be rate-determining.

It is clear that there are significant differences between energy barriers at different levels of theory, but at this point, we cannot ensure which DF performs best against the available experimental data. One possibility to assess the accuracy of the DF would be to perform post-HF calculations, such as DLPNO–CCSD(T),^73^ but our system is relatively large in terms of the number of atoms and the number of species involved in the mechanism. Indeed, only a few studies report the use of this method on selected MOF cluster structures.^74,75^ There is a recent study on transition metal barrier heights at DLPNO–CCSD(T) and DFT levels,^76^ but no reactions involving Zr were included in the database.

Thus, we decided to benchmark our DFT results against experimental data. We employ these DFT-computed electronic energies and Hessian matrices to predict reaction rates via a KMC approach.

### Reaction Kinetics Simulations and Comparison with Experiments

We first selected data from the M06 Gibbs energy profile. The KMC simulation was applied to the four main steps defined by the following reactions:
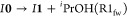

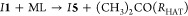

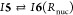

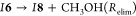

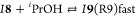

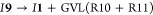

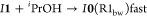
Notice that between **I1** and **I5** there are several equilibrium reactions (R2–R5) with just one transition state **TS2–3**, which corresponds to the hydrogen transfer reaction plus the release of acetone. They were collected in a reaction named *R*_HAT_. Reaction *R*_nuc_ (or R6) is the nucleophilic attack through transition state **TS5–6**; *R*_elim_ is the elimination reaction through transition state **TS6–7** and comprises reactions R7 and R8. Of the two of them, R7 is the rate-determining step. The regeneration step comprises reactions from R9 to R1 where transition **TS9–10** has the highest Gibbs energy in this last set of reactions.

One of the experiments^9^ (hereafter, experiment 1) was carried out at *T* = 413.15 K and 100 mg (0.057 mmol) of catalyst. At that temperature, the bimolecular rate constant for reaction *R*_HAT_ evaluated by MS-TST at the M06 level is 1.34 × 10^–15^ cm^3^ molecule^–1^ s^–1^. The concentration of ML is 0.2 M (1 mmol of ML in 5 mL of alcohol), leading to a pseudo-first-order rate constant for the hydrogen transfer reaction of 1.61 × 10^5^ s^–1^. In this case, we have employed MS-TST instead of TST because ML presents several torsional conformers as a reactant, being the latest a factor of 2.20 larger than the former.

The forward and reverse nucleophilic attack unimolecular rate constants evaluated employing TST are 7.08 × 10^2^ and 2.54 × 10^10^ s^–1^, respectively. The rate constant for the elimination step is 1.35 × 10^6^ s^–1^. The reverse rate constant for the nucleophilic attack is much faster than its forward counterpart and that of the elimination reaction.

This makes it possible to assemble *R*_nuc_ and *R*_elim_ in one reaction,
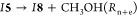
where *R*_n+e_ incorporates the nucleophilic attack and elimination steps. The value of the rate constant for these two combined reactions is 3.75 × 10^–2^ s^–1^. This value was obtained by CVT theory, which, due to the variational effects, is 1.15 times lower than the value calculated by TST.

As to the regeneration step, both R9 and R1_bw_ bimolecular reactions that involve the participation of ^*i*^PrOH are fast because this alcohol is in excess. Reaction *R*_reg_ through **TS9–10** was evaluated by TST and the rate constant is 1.87 × 10^9^ s^–1^, whereas the release of ^*i*^PrOH (R1_fw_) is a barrierless reaction with an estimated value of the rate constant of 8.64 × 10^5^ s^–1^ at 413 K. This rate constant was calculated considering that the Gibbs energy of activation is the same as the one at products. Therefore, the regeneration is much faster than the nucleophilic attack and elimination and can be ignored.

The simulation of the reaction network by KMC leads to a reaction time that is fully in agreement with the reduced mechanism that just contains the *R*_HAT_ and *R*_n+e_ reactions. Under this assumption, it is possible to reduce the reaction time, *t*_α_ (for a given product conversion, α), to an analytical expression of the type (see the Supporting Information, SI, for details):
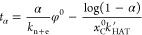
1where *k*_HAT_^′^ is the ^*i*^PrOH release plus the hydrogen transfer, *k*_n+e_ is the nucleophilic attack plus elimination rate constants, and φ^0^ is the ratio between the initial concentrations of ML, *x*_ML_^0^, and the catalyst, *x*_C_^0^. Considering that *x*_C_^0^*k*_HAT_^′^ ≫ *k*_n+e_/φ^0^, or similarly that *x*_ML_^0^*k*_HAT_^′^ ≫ *k*_n+e_, eq 1 reduces to
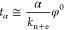
2A consequence of having the second reaction as rate-determining invalidates any influence of the multiple conformations of methyl or ethyl levulinate, which, on the other hand, have a very modest contribution.

Under the experimental conditions previously indicated,^9^ φ_exp1_^0^ ≈ 18. However, the theoretical calculations by applying eq 1 predict a value of φ^0^ = 1734 for the reaction time of the experiment (9 h) and α = 0.70.

A different experiment carried out by Valekar et al.^8^ (hereafter, experiment 2) at *T* = 403.15 K involved a reaction time of 3 h, and the conversion of the reactant (in this case ethyl levulinate) was 43% with a ratio φ_exp2_^0^ ≈ 35. In this case, *k*_n+e_ = 1.80 × 10^–2^ s^–1^ leading to a calculated value of φ^0^ = 453.

In both cases, the ratio between the calculated and experimental φ^0^ is larger than 1. Specifically, it is about 100 for experiment 1 and about 10 for experiment 2. The theoretical results are very encouraging, taking into account the complexity of the reaction mechanism and the catalyst structure.

Here, we point out some possible reasons for the discrepancy between theory and experiment. First, we know the concentration of the MOF catalyst, but we do not know how many active sites (i.e., defects) are available under these conditions. If there are inoperative active sites, the ratio φ_exp_^0^ would be larger. Related to this, eq 1 predicts a linear relation between the reaction time and the conversion; this may be true when α is small, but the variation is less acute at large α values. Thus, the experimental reaction time that we introduce in eq 1 overestimates the theoretical value of φ^0^.

Second, the DFT calculations may have overestimated the value of *k*_n+e_. At 400 K, an increase of 2 kcal·mol^–1^ in the barrier height amounts to a decrease of more than an order of magnitude in the thermal rate constant. As discussed before, the choice of the DF has a significant impact. The best results are obtained with M06, while the rest of the DFs overestimate φ^0^ substantially. For PBE-D3, *k*_n+e_ = 2.35 × 10^+2^ s^–1^ and *k*_n+e_ = 1.40 × 10^+2^ s^–1^ for experiments 1 and 2, respectively. These values are at least 4 orders of magnitude larger than the M06 results. Consequently, the calculated values for φ^0^ employing eq 2 and the PBE-D3 rate constants are very far from the experimental results. However, the rate-determining step is the same as for the M06 calculations, that is, the nucleophilic attack plus elimination reactions.

Finally, we have ignored the role of the diffusion inside the MOF pores by assuming that it is a relatively fast process and the fact that there are side reactions that compete with the GVL formation (e.g., transesterification) and that may affect the availability of the active sites. Despite all of the limitations of the mechanism proposed, the combination of the M06 level with KMC calculations has proved to be a useful computational tool to address reactions involving MOF-based systems.

## Conclusions

Here, we present a detailed computational analysis of a MOF-catalyzed case study reaction, the conversion of ML to GVL via UiO-66, by evaluating different models and levels of theory as well as comparing computed kinetic data with experiments. We found that (i) cluster models follow the same trend as periodic ones, presenting nearly identical geometries and similar activation barriers; (ii) the DF has a strong impact on Gibbs energy barriers, especially those involving a hydrogen transfer process; and (iii) the KMC analysis reveals the reaction rate equations, where M06 is the best-performing method with a reasonable good agreement with experimental results, but PBE greatly overestimates rate constants. Due to the lack of benchmark studies for MOF-based systems, we expect that the present data would be useful for other MOF-catalyzed processes.

## Supporting Information Available

Deduction of eq 1, additional details about the thermal rate constants calculations, electronic energy profiles, geometrical parameters of interest, and conformers of methyl levulinate (PDF)
