# Supplementary material for: Combined DFT and Kinetic Monte Carlo Study of UiO-66 Catalysts for γ-Valerolactone Production
Source: J Phys Chem C Nanomater Interfaces. 2024 Jan 12;128(3):1049–57. doi: 10.1021/acs.jpcc.3c06053 (PMC10823797; doi:10.1021/acs.jpcc.3c06053)
Supplement: Supplementary file 1 — jp3c06053_si_001.pdf [file jp3c06053_si_001.pdf]

Supporting Information for

Combined DFT and Kinetic Monte Carlo Study of UiO-66 Catalysts for γ-Valerolactone Production

Thanh-Hiep Thi Le[a], David Ferro-Costas[a,b], Antonio Fernández-Ramos*[a,b], and Manuel A. Ortuño*[a]

[a]Centro Singular de Investigación en Química Biolóxica e Materiais Moleculares (CIQUS), Universidade de Santiago de Compostela, 15782, Santiago de Compostela, Spain
[b]Departamento de Química Física, Facultade de Química, Universidade de Santiago de Compostela, 15782 Santiago de Compostela, Spain

Contents

1 Analytical Expression for the Reaction Time **S2**

2 Calculation of thermal rate constants **S3**

3 Electronic Energy Reaction Profiles **S5**
3.1 Cluster PBE-D2 level *versus* periodic PBE-D2 . . . . . S5
3.2 Cluster PBE-D3 versus cluster PBE-D3 with a diffuse function basis set for O atoms S6

4 Different routes for regeneration **S7**

5 Distances of interest **S8**

6 Conformers of Methyl Levulinate **S10**
6.1 Reference Z-matrix S10
6.2 Input file for `TorsiFlex` S11
6.3 Target torsions S12
6.4 Conformers S13
6.5 Cartesian coordinates S14

*qf.ramos@usc.es, manuelangel.ortuno@usc.es

S1. Analytical Expression for the Reaction Time

The reaction mechanism under investigation can be conveniently expressed as:

$$\begin{array}{ccc} R+C & \xrightarrow{k_1} & I \\ I & \xrightarrow{k_2} & P+C \end{array} \tag{S1}$$

in order to estimate the reaction time (see main manuscript). In it, R and P represent the reactant (methyl levulinate) and the product (γ-valerolactone), respectively, whereas C is the catalyst and I is a reaction intermedium. Notice that

$$x_{\mathrm{I}} = x_{\mathrm{C}}^{0} - x_{\mathrm{C}} \tag{S2}$$

where x is the concentration of a given species, indicated by a subscript, and the 0 superscript indicates the initial concentration.

From the previous mechanism, we can write:

$$\frac{d}{dt}x_{\mathrm{R}} = -k_1 \cdot x_R \cdot x_{\mathrm{C}} \tag{S3}$$

$$\frac{d}{dt}x_{\mathrm{I}} = +k_1 \cdot x_{\mathrm{R}} \cdot x_{\mathrm{C}} - k_2 \cdot x_{\mathrm{I}} \tag{S4}$$

If we assume the steady state approximation, *i.e.* the intermediate is consumed as quickly as it is generated, in combination with eq. (S2), we get:

$$\frac{d}{dt}x_{\mathrm{I}} \simeq 0 \rightarrow k_2 \cdot x_{\mathrm{I}} \simeq k_1 \cdot x_{\mathrm{R}} \cdot x_{\mathrm{C}} \rightarrow x_{\mathrm{C}} = \frac{k_2}{k_2 + k_1 \cdot x_{\mathrm{R}}} x_{\mathrm{C}}^{0} \tag{S5}$$

Thus, integrating eq. (S3):

$$\int_{x_{\mathrm{R}}^{0}}^{x_{\mathrm{R}}} \left(\frac{k_2}{x_{\mathrm{R}}} + k_1 \right) dx_{\mathrm{R}} = -k_1 k_2 x_{\mathrm{C}}^{0} \int_{0}^{t_\alpha} dt \tag{S6}$$

with t_α being the time at which $x_{\mathrm{R}} = (1-\alpha) \cdot x_{\mathrm{R}}^{0}$, lead us to:

$$t_\alpha = \frac{\alpha}{k_2}\varphi^{0} - \frac{\ln(1-\alpha)}{x_{\mathrm{C}}^{0} \cdot k_1} \tag{S7}$$

where α is the conversion. In eq (S7), φ^0 represents the ratio between the initial populations of the reactant and the catalyst:

$$\varphi^{0} = x_{\mathrm{R}}^{0} / x_{\mathrm{C}}^{0} \tag{S8}$$

S2. Calculation of thermal rate constants

To simulate the UiO-66 MOF's rigidity, the four *para*-carbon atoms of the benzoate groups were kept fixed during the calculations. As a result, the systems with frozen atoms (**I0** to **I10** and the transition states) will contribute with a total of $3 \cdot (N-4)$ vibrational modes to the partition function, where N represents the total number of atoms. Notably, the overall translations and rotations are excluded in the calculation of the partition function. It is essential to mention that the `Pilgrim` software used for these calculations was not originally designed for systems with frozen atoms. To overcome this limitation, a modified version of `Pilgrim` was employed for the calculation of the rate constants.

Rate constants were calculated using several DFT (Density Functional Theory) functionals. Hessian matrices were calculated using the Stuttgart effective core potentials (SDD) in combination with the def2-TZVP basis set for the Zr atoms and def2-SVP for O, C, and H atoms. A cutoff of 50 cm^{-1} was implemented for the resulting vibrational frequencies. The electronic energies were refined using single-point calculations using the def2-TZVP/SDD basis set for Zr and def2-TZVP for the O, C, and H atoms.

The calculated rate constants and ratios between the initial concentration of **ML** and the catalyst, φ^0, can be found in Tables S1 and S2. We highlight that:

- Multi-structural transition state theory (MS-TST) was used in the calculation of the rate constant of $\mathrm{R_{HAT}}$, in order to account for the conformational wealth of **ML** (see section S6):

$$k^{\mathrm{MS-TST}} = \left(\frac{F^{\mathrm{MS},\ddagger}}{F^{\mathrm{MS,R}}}\right) \cdot k^{\mathrm{SS-TST}} = F^{\mathrm{MS}} \cdot k^{\mathrm{SS-TST}} \tag{S9}$$

  In the previous equation, $k^{\mathrm{SS-TST}}$ is the single-structure TST rate constant and $F^{\mathrm{MS,X}}$ [X = ‡ (the TS), or R (reactants)] accounts for the contribution of the conformations of **ML**, that is, it is the ratio between the MS and SS harmonic-oscillator partition functions. The rate constant $k^{\mathrm{SS-TST}}$ was calculated for each DFT functional, but F_{MS} was exclusively calculated for M06 and assumed to be the same for M06L anf PBE-D3. The value of F^{MS} at $T = 413$ K is 0.46.

- Forward and backward rate constants for $\mathrm{R_{nuc}}$ were calculated using SS-TST.

- The rate constant for $\mathrm{R_{elim}}$ (and consequently for $\mathrm{R_{n+e}}$) was calculated using canonical variational transition state theory (CVT):

$$k^{\mathrm{SS-CVT}} = \Gamma^{\mathrm{CVT}} \cdot k^{\mathrm{SS-TST}} \tag{S10}$$

  The variational coefficient, Γ^{CVT}, associated with **TS6-7** is constant between 403.15 and 413.15 K and its value is 0.87, 0.38 and 0.70 for M06, M06L and PBE-D3, respectively.

Table S1: Rate constants calculated by different electronic structure methods for the reactions indicated in the "Reaction kinetics simulations and comparison with experiments" section. Rate constants are in $\mathrm{cm^3 \cdot molecule^{-1} \cdot s^{-1}}$ for $\mathrm{R_{HAT}}$ and in $\mathrm{s^{-1}}$ for the remaining reactions. Forward and backward rate constants are indicated with (fw) and (bw), respectively.

Reaction	Experiment 1 (413.15 K)			Experiment 2 (403.15 K)		
	M06	M06L	PBE-D3	M06	M06L	PBE-D3
R1 (fw)	8.64E+05	1.55E+05	5.18E+05	3.52E+05	5.98E+04	2.08E+05
$\mathrm{R_{HAT}}$	1.34E-15	2.86E-13	4.95E-12	1.90E-15	4.75E-13	8.25E-12
$\mathrm{R_{nuc}}$ (fw)	7.08E+02	7.58E+03	1.23E+04	4.24E+02	4.63E+03	7.78E+03
$\mathrm{R_{nuc}}$ (bw)	2.54E+10	5.58E+11	2.36E+11	2.10E+10	4.93E+11	2.03E+11
$\mathrm{R_{elim}}$	1.35E+06	3.80E+07	4.53E+09	8.91E+05	2.79E+07	3.66E+09
$\mathrm{R_{n+e}}$	3.75E-02	5.16E-01	2.35E+02	1.80E-02	2.62E-01	1.40E+02
$\mathrm{R_{reg}}$	1.87E+09	2.19E+09	1.72E+11	1.59E+09	1.81E+09	1.59E+11

Table S2: Experimental (Exp) and calculated values by different electronic structure methods for the ratio between the initial concentrations of ML and the catalyst (φ^0). The value of the product conversion (α) for each experiment is also shown.

		φ^0			
	α	Exp	M06	M06L	PBE-D3
Experiment 1	0.70	18	1 734	23 865	10 873 630
Experiment 2	0.43	35	453	6 576	3 525 112

S3. Electronic Energy Reaction Profiles

S3.1. Cluster PBE-D2 level *versus* periodic PBE-D2

Figure S1 shows the electronic energy profiles for cluster and periodic models computed at PBE level with Grimme dispersion D2 (while in Figure 3, cluster PBE-D3 was compared with periodic PBE-D2). The dispersion correction does not affect much the activation energies of the reaction.

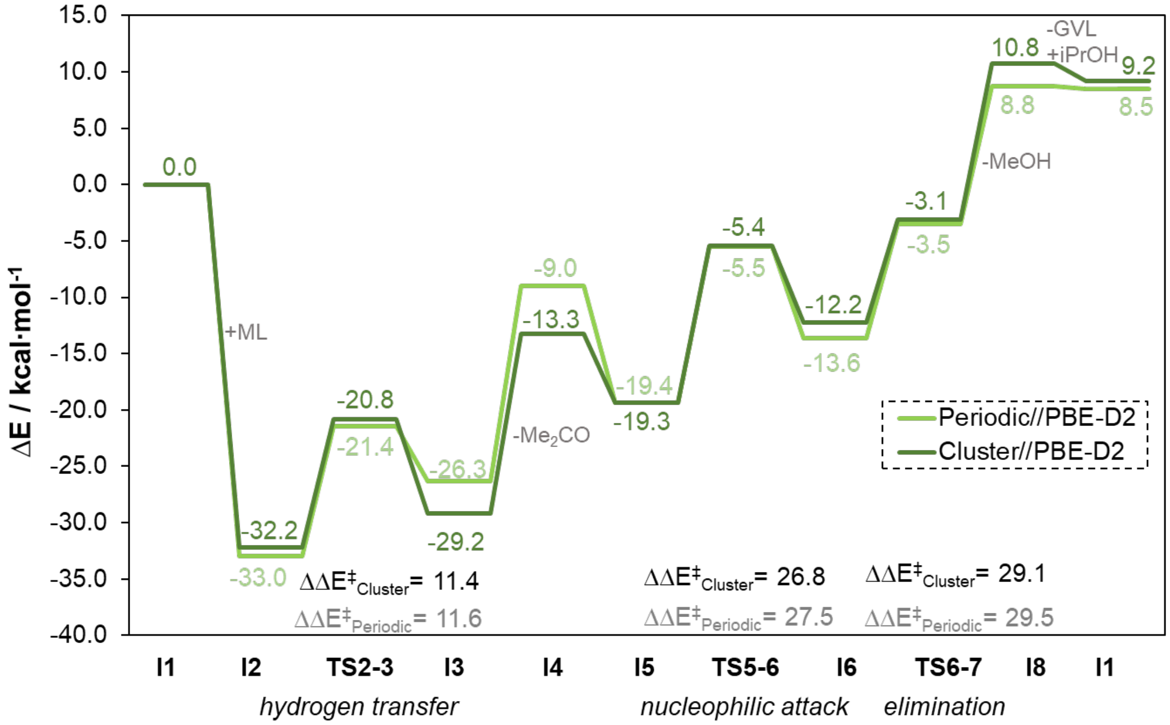

Figure S1: Electronic energy profiles (in kcal $\cdot mol^{-1}$) of defective UiO-66 using cluster at the PBE-D2 level and periodic at the PBE-D2 level. R = $(CH_2)_2CO_2Me$

S3.2. Cluster PBE-D3 versus cluster PBE-D3 with a diffuse function basis set for O atoms

To better represent electronegative atoms with the basis set, we employed diffuse functions for all oxygen (O) atoms and referred to this modified basis set as "basis set diffuse". The results, shown in Figure S2, indicate that the inclusion of diffuse functions does not significantly impact the activation energies of the reaction.

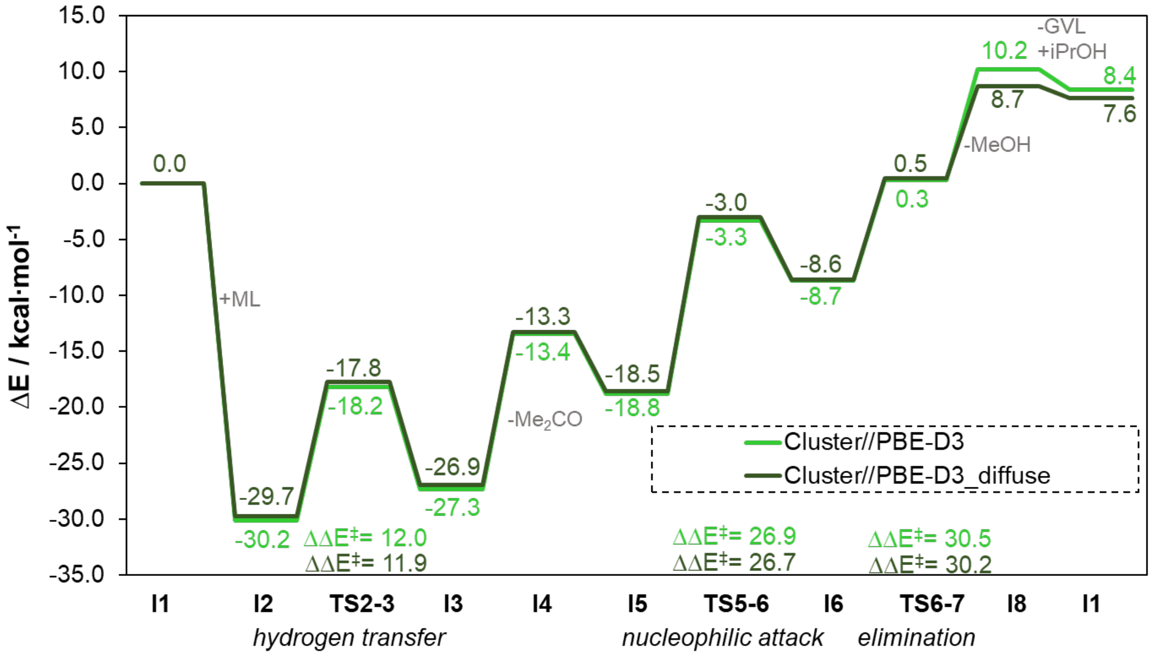

Figure S2: Electronic energy profiles (in kcal·mol^{-1}) of defective UiO-66 using cluster at the PBE-D3 level and at the PBE-D3 level with a diffuse function basis set for O atoms. R = $(CH_2)_2CO_2Me$.

S4. Different routes for regeneration

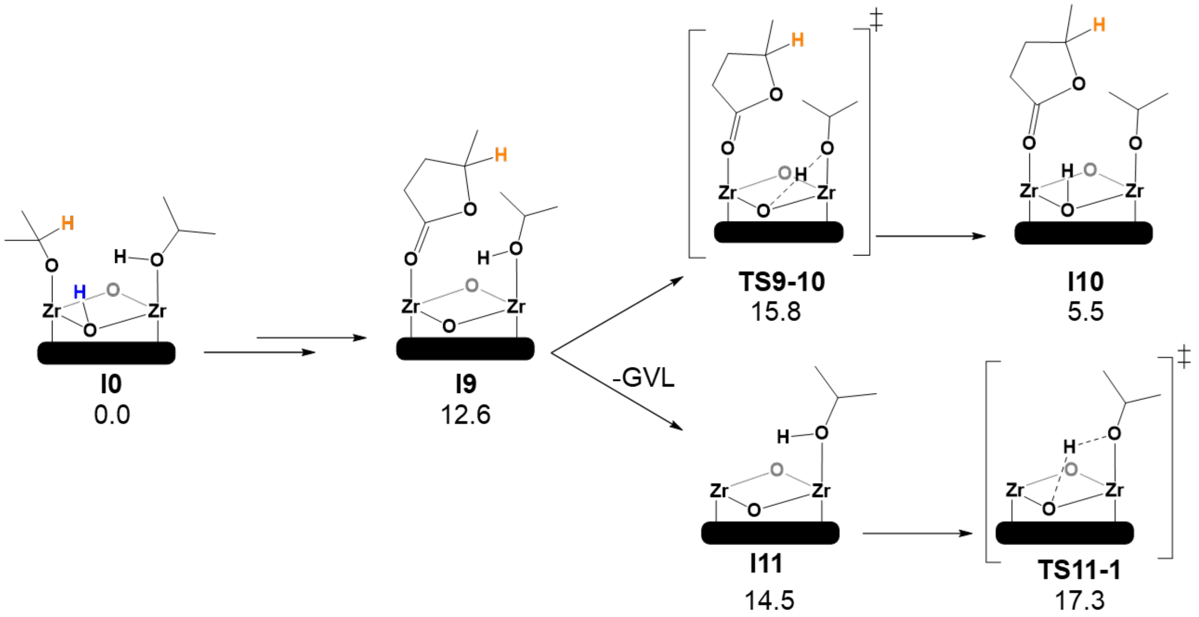

Figure S3: Relative Gibbs energies calculated at the PBE-D3 level for the regeneration routes at 403.15 K (in kcal·mol^{-1}).

S5. Distances of interest

Table S3: Computed geometries of intermediates and transition states across different DFs. Selected distances in Å.

Computed species		PBE-D3	M06-L	PBE0-D3	M06
**I0**	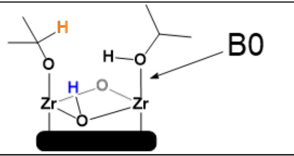	B0 = 2.272	B0 = 2.299	B0 = 2.257	B0 = 2.299
**I1**	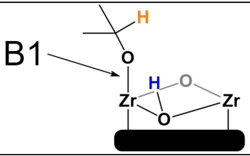	B1 = 1.966	B1 = 1.969	B1 = 1.952	B1 = 1.959
**I2**	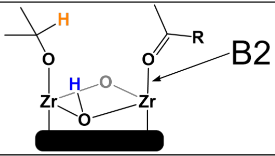	B2 = 2.228	B2 = 2.280	B2 = 2.264	B2 = 2.274
**TS2-3**	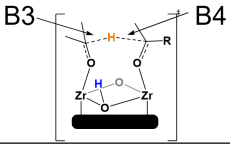	B3 = 1.314<br>B4 = 1.370	B3 = 1.296<br>B4 = 1.349	B3 = 1.295<br>B4 = 1.357	B3 = 1.310<br>B4 = 1.357
**I3**	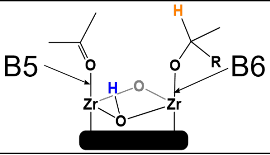	B5 = 2.290<br>B6 = 1.991	B5 = 2.294<br>B6 = 1.992	B5 = 2.265<br>B6 = 1.971	B5 = 2.292<br>B6 = 1.982
**I4**	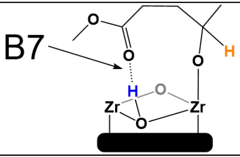	B7 = 1.681	B7 = 1.743	B7 = 1.678	B7 = 1.745
**I5**	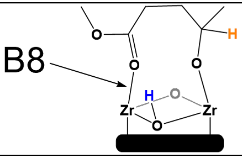	B8 = 2.315	B8 = 2.299	B8 = 2.287	B8 = 2.313
**TS5-6**	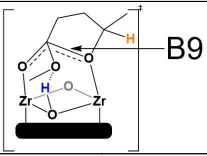	B9 = 2.176	B9 = 2.105	B9 = 2.127	B9 = 2.060
**I6**	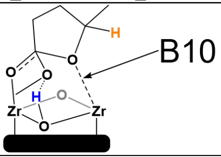	B10 = 2.402	B10 = 2.377	B10 = 2.379	B10 = 2.397

**TS6-7**	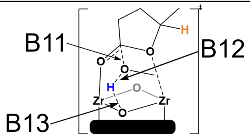	B11 = 1.945<br>B12 = 1.178<br>B13 = 1.258	B11 = 2.007<br>B12 = 1.273<br>B13 = 1.148	B11 = 1.889<br>B12 = 1.124<br>B13 = 1.299	B11 = 1.958<br>B12 = 1.192<br>B13 = 1.214
**I7**	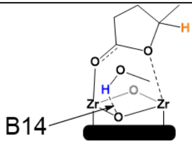	B14 = 1.759	B14 = 1.790	B14 = 1.747	B14 = 1.832
**I8**	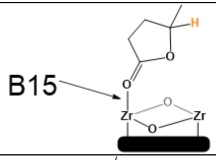	B15 = 2.384	B15 = 2.381	B15 = 2.355	B15 = 2.375
**I9**	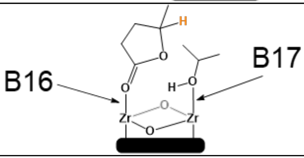	B16 = 2.460<br>B17 = 2.355	B16 = 2.411<br>B17 = 2.354	B16 = 2.420<br>B17 = 2.325	B16 = 2.436<br>B17 = 2.332
**TS9-10**	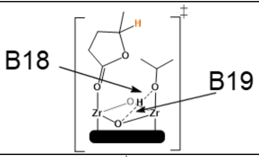	B18 = 1.178<br>B19 = 1.275	B18 = 1.180<br>B19 = 1.265	B18 = 1.171<br>B19 = 1.255	B18 = 1.147<br>B19 = 1.287
**I10**	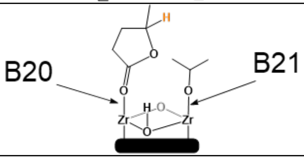	B20 = 2.354<br>B21 = 1.968	B20 = 2.341<br>B21 = 1.974	B20 = 2.324<br>B21 = 1.957	B20 = 2.334<br>B21 = 1.966

S6. Conformers of Methyl Levulinate

S6.1. Reference Z-matrix

Here, we show the Z-matrix used in the conformational search with `TorsiFlex`.

```
C
C   1   dist02
C   2   dist03   1   angl03
C   3   dist04   2   angl04    1   ptor04
C   4   dist05   3   angl05    2   ptor05
O   5   dist06   4   angl06    3   ptor06
C   6   dist07   5   angl07    4   ptor07
O   2   dist08   1   angl08    3   itor08
O   5   dist09   4   angl09    6   itor09
H   7   dist10   6   angl10    5   ptor10
H   1   dist11   2   angl11    3   ptor11
H   7   dist12   6   angl12   10   itor12
H   7   dist13   6   angl13   10   itor13
H   4   dist14   3   angl14    5   itor14
H   4   dist15   3   angl15    5   itor15
H   3   dist16   2   angl16    4   itor16
H   3   dist17   2   angl17    4   itor17
H   1   dist18   2   angl18   11   itor18
H   1   dist19   2   angl19   11   itor19
```

The value associated to each internal coordinate is listed below. Bond distances are given in Å; bond and dihedral angles, in degrees.

```
dist02 =    1.49049
dist03 =    1.49364
angl03 =  122.49417
dist04 =    1.51886
angl04 =  107.28266
ptor04 =  123.26838
dist05 =    1.47337
angl05 =  110.40500
ptor05 = −179.64980
dist06 =    1.37991
angl06 =  123.80413
ptor06 =  178.44275
dist07 =    1.40100
angl07 =  124.10743
ptor07 = −179.86029
dist08 =    1.22272
angl08 =  118.03470
itor08 =  179.91647
dist09 =    1.25956
angl09 =  119.38251
itor09 =  179.87802
dist10 =    1.12518
angl10 =  113.30641
ptor10 =  −43.29074
dist11 =    1.10083
angl11 =  108.38207
ptor11 =  109.29496
dist12 =    1.09791
angl12 =  113.83050
itor12 =  122.44000
dist13 =    1.12056
angl13 =  109.45308
itor13 = −114.38634
dist14 =    1.12320
angl14 =  108.59431
itor14 =  122.04823
dist15 =    1.10139
angl15 =  107.09845
itor15 = −123.56398
dist16 =    1.09873
angl16 =  106.63132
itor16 =  120.44454
dist17 =    1.10312
angl17 =  109.78956
itor17 = −123.69079
dist18 =    1.10934
angl18 =  107.76658
itor18 =  117.14249
dist19 =    1.11319
angl19 =  109.21210
itor19 = −124.02363
```

S6.2. Input file for `TorsiFlex`

```
#-----------------------------#
#          System             #
#-----------------------------#
zmatfile     ml.zmat          # Z-matrix file
charge       0                # charge of the system
multipl      1                # multiplicity of the system
enantio      yes              # yes if torsional enantioners, no otherwise
ts           no               # yes if transition state, no otherwise
cfactor      1.3              # controls the connectivity criterium

#-----------------------------#
#          Storage            #
#-----------------------------#
dirll  files_LL/              # folder to store LL conformers
dirhl  files_M06/             # folder to store HL conformers
tmpll  /scratch/LL_ml/        # folder for LL temporal files
tmphl  /scratch/M06_ml/       # folder for HL temporal files

#-----------------------------#
#      Target torsions        #
#-----------------------------#
torsion1     ptor04
torsion2     ptor05
torsion3     ptor06
torsion4     ptor07
precond4     0 180

#-----------------------------#
#     Search  Procedure       #
#-----------------------------#
ncycles      100              # number of steps of stochastic algorithm

#-----------------------------#
#     Validation  tests       #
#-----------------------------#
testsG       1 1 1 1          # for Guess geom (Conn, Simil, Hard, Soft)
testsO       1 1 1 1          # for Opt   geom (Conn, Redun, Hard, Soft)
dist1D       7                # domain size about each point (degrees)
epsdeg       2                # max diff between two identical angles (degrees)

#-----------------------------#
# Gaussian calculations       #
#-----------------------------#
optmode      1                # 0:opt(z-matrix) , 1:opt(modredundant)
fccards      yes              # Use LL Hessian in HL opt (yes/no)
nprocll      8                # Number of threads ( low-level)
nprochl      8                # Number of threads (high-level)
memll        16GB             # dynamic memory ( low-level)
memhl        16GB             # dynamic memory (high-level)

lowlevel     HF    3-21G                    #  low-level of calculation
highlevel    M06 Def2SVP  int=ultrafine # high-level of calculation

#-----------------------------#
#   Partition functions       #
#-----------------------------#
temps        403.15           # temperatures (K) for part. functions
temps        413.15           # temperatures (K) for part. functions
freqscalLL   1.000            # freq. scaling factor (LL)
freqscalHL   1.000            # freq. scaling factor (HL)
sigmamj      0.02             # max value for sigma(Mj); >= 0.01
```

S6.3. Target torsions

The following torsions were selected as target torsions for the conformational search:

- ϕ_1: C1-C2-C3-C4 (`ptor04`)
- ϕ_2: C2-C3-C4-C5 (`ptor05`)
- ϕ_3: C3-C4-C5-O6 (`ptor06`)
- ϕ_4: C4-C5-O6-C7 (`ptor07`)

The name of the torsion (between brackets) corresponds to the one used in the definition of the Z-matrix. Notice that torsions associated to rotation of methyl groups (`ptor10` and `ptor11`) are excluded, as they do not lead to new conformers.

S6.4. Conformers

Relative energy for the conformers for methyl levulinate, calculated at M06/Def2SVP, is shown in Table S4. The two reference energies, one for the total electronic energy (U) and another for the total electronic energy plus the vibrational zero-point energy (U+ZPE), are:

- U_{ref} : -459.72543 Hartree
- $(U + \mathrm{ZPE})_{\mathrm{ref}}$: -459.57003 Hartree

Notice that each conformer in Table S4 presents a torsional enantiomer. Therefore, the total number of located conformers is twice the number shown in the table.

Table S4: Relative total energy, U, and relative total energy plus zero-point energy, $U + \mathrm{ZPE}$, for the located conformers of methyl methyl levulinate (kcal/mol). Dihedral angles are in degrees.

Conf	$(\phi_1, \phi_2, \phi_3, \phi_4)$	U	$U + \mathrm{ZPE}$
1	(147,293,183,181)	0.000	0.000
2	(175,177,177,179)	1.585	1.038
3	(186,300,330,179)	1.838	1.711
4	(155,292,002,178)	1.840	1.741
5	(170,174,322,182)	3.113	2.769
6	(044,281,155,177)	3.457	3.722
7	(323,299,200,182)	3.466	3.342
8	(317,293,034,180)	4.222	4.152
9	(314,300,276,176)	4.254	4.405
10	(314,071,047,180)	4.440	4.522
11	(315,183,319,181)	5.658	5.533
12	(147,293,180,358)	6.969	6.962
13	(174,294,141,346)	7.455	7.419
14	(177,179,180,000)	8.464	7.921
15	(316,084,184,000)	11.170	11.043
16	(033,061,170,359)	11.269	11.026
17	(045,060,090,004)	12.701	12.907
18	(064,036,069,351)	13.037	13.048
19	(060,275,282,347)	14.109	14.097

S6.5. Cartesian coordinates

In this section, the Cartesian coordinates (in Å) of each conformer optimized at the M06/Def2SVP level are listed.

```
19
 * E =  +0.000 kcal/mol ; (1) 147_293_183_181
 C    +0.00000000    +0.00000000    +0.00000000
 C    +0.00000000    +0.00000000    +1.49820379
 C    +1.36435470    +0.00000000    +2.16446707
 C    +1.34335524    +0.76070333    +3.46953808
 C    +1.10570588    +2.22422564    +3.22637868
 O    +1.13740613    +2.92280947    +4.36475211
 C    +0.89369232    +4.31325663    +4.25062565
 O    −1.01407323    +0.00327182    +2.15501022
 O    +0.90668855    +2.72034041    +2.14641481
 H    −0.09988872    +4.50446140    +3.81650120
 H    +0.72205941    −0.72739023    −0.40508927
 H    +0.94651254    +4.72786879    +5.26446001
 H    +1.64610009    +4.79507392    +3.60694556
 H    +0.52468135    +0.39524736    +4.11292554
 H    +2.27194147    +0.64321277    +4.05177899
 H    +1.63385525    −1.05818348    +2.33857257
 H    +2.12215743    +0.40692214    +1.47410196
 H    −1.00784697    −0.19561890    −0.38895199
 H    +0.33227858    +1.00045497    −0.32723544
19
 * E =  +1.585 kcal/mol ; (2) 175_177_177_179
 C    +0.00000000    +0.00000000    +0.00000000
 C    +0.00000000    +0.00000000    +1.50462321
 C    +1.35945743    +0.00000000    +2.15959964
 C    +1.30236803    +0.13047765    +3.66168597
 C    +2.65542055    +0.05304765    +4.30376581
 O    +2.56976034    +0.24253521    +5.62718464
 C    +3.78793491    +0.17971109    +6.34464253
 O    −1.02183200    +0.00532227    +2.15009539
 O    +3.69341016    −0.15481850    +3.73107805
 H    +4.49623097    +0.94500692    +5.99041237
 H    +0.68286912    −0.76705323    −0.39894887
 H    +3.54369956    +0.35468482    +7.39939519
 H    +4.26633959    −0.80535153    +6.22779330
 H    +0.81815629    +1.07273878    +3.97069775
 H    +0.66090062    −0.65210622    +4.10284058
 H    +1.88323531    −0.93005290    +1.86804153
 H    +1.97803956    +0.79811172    +1.70834322
 H    −1.01763555    −0.16302054    −0.37832394
 H    +0.36892544    +0.97234581    −0.36763331
19
 * E =  +1.838 kcal/mol ; (3) 186_300_330_179
 C    +0.00000000    +0.00000000    +0.00000000
 C    +0.00000000    +0.00000000    +1.50313704
 C    +1.35702826    +0.00000000    +2.16894970
```

C +1.26889695 −0.14099543 +3.67264142
C +0.50332578 +0.94746105 +4.37728890
O +0.58926067 +2.12029999 +3.72610941
C −0.12299596 +3.19612634 +4.30655877
O −1.01997096 −0.00094978 +2.15134770
O −0.07091534 +0.81525476 +5.42255203
H +0.23551508 +3.40565134 +5.32646108
H +0.41428015 −0.95133362 −0.37376095
H +0.04115723 +4.06991287 +3.66389814
H −1.19845518 +2.96638420 +4.36232464
H +0.78790321 −1.08780916 +3.96258749
H +2.28147942 −0.15711763 +4.11248300
H +1.96927752 −0.80706322 +1.72666001
H +1.86595934 +0.93970529 +1.88612255
H −1.02326451 +0.12205693 −0.37847365
H +0.64661590 +0.80171125 −0.39180194
19
* E = +1.840 kcal/mol ; (4) 155_292_002_178
C +0.00000000 +0.00000000 +0.00000000
C +0.00000000 +0.00000000 +1.50052117
C +1.36562001 +0.00000000 +2.15833586
C +1.33894343 +0.59092633 +3.54937762
C +1.06707287 +2.06978524 +3.62134900
O +0.85342022 +2.61249111 +2.40913215
C +0.54783381 +3.99523625 +2.39862739
O −1.01598733 −0.00241200 +2.15340578
O +1.04225014 +2.70768064 +4.63805114
H +1.36868817 +4.58334912 +2.83733542
H +0.72421038 −0.72649752 −0.40294812
H +0.39883306 +4.27980758 +1.34995480
H −0.36582713 +4.19881063 +2.97783016
H +0.54611221 +0.10973099 +4.14669786
H +2.27985655 +0.41375595 +4.09498520
H +1.68956689 −1.05741648 +2.20161965
H +2.09485583 +0.50962218 +1.50591077
H −1.00697911 −0.20310467 −0.38755399
H +0.32723259 +0.99755862 −0.34118770
19
* E = +3.113 kcal/mol ; (5) 170_174_322_182
C +0.00000000 +0.00000000 +0.00000000
C +0.00000000 +0.00000000 +1.50424898
C +1.36167593 +0.00000000 +2.15888429
C +1.30229888 +0.24182718 +3.65179707
C +2.61218493 +0.12444927 +4.37614517
O +3.36535011 −0.87706966 +3.88735245
C +4.60212036 −1.09631943 +4.53995885
O −1.02018679 −0.00226533 +2.15089868
O +2.95710596 +0.80845694 +5.30153060
H +4.44998123 −1.34544952 +5.60172644
H +0.73398221 −0.71617357 −0.40256691
H +5.08881526 −1.93323757 +4.02441216
H +5.24010228 −0.20023166 +4.49032517

H +0.88401642 +1.23197256 +3.88782342
H +0.60368476 −0.48489422 +4.10420208
H +1.82775478 −0.97937009 +1.94130940
H +2.00894889 +0.73488212 +1.64431757
H −1.00487112 −0.22747622 −0.37932986
H +0.29918515 +0.99823524 −0.36219503
19
* E = +3.457 kcal/mol ; (6) 044_281_155_177
C +0.00000000 +0.00000000 +0.00000000
C +0.00000000 +0.00000000 +1.50574136
C +1.34741154 +0.00000000 +2.20110985
C +2.37783190 +0.95741376 +1.62643369
C +3.05143775 +0.44500124 +0.38221081
O +3.50672644 +1.45149686 −0.37250611
C +4.21345945 +1.08156770 −1.54427545
O −1.02509200 −0.02813681 +2.14361267
O +3.19302874 −0.71513696 +0.09486251
H +3.57425940 +0.49088565 −2.21867736
H +0.58264256 −0.85435056 −0.38146555
H +4.51919975 +2.01283862 −2.03566995
H +5.09920386 +0.47648097 −1.29648282
H +1.96694144 +1.96187461 +1.43014428
H +3.18805403 +1.11551097 +2.36111919
H +1.15479345 +0.20588539 +3.26436357
H +1.75147188 −1.02762900 +2.13244883
H −1.03367703 −0.05072104 −0.36653947
H +0.47977675 +0.91061164 −0.39910492
19
* E = +3.466 kcal/mol ; (7) 323_299_200_182
C +0.00000000 +0.00000000 +0.00000000
C +0.00000000 +0.00000000 +1.50549346
C +1.34682827 +0.00000000 +2.20737393
C +2.46694625 −0.80057541 +1.57217293
C +2.14587874 −2.26521668 +1.47650682
O +3.24945447 −3.00300027 +1.32082939
C +3.05065436 −4.39886837 +1.17405513
O −1.02044171 +0.06751799 +2.14483301
O +1.03453060 −2.72736814 +1.50710463
H +2.43001199 −4.61843584 +0.29146238
H +0.27369801 −1.00178963 −0.36880470
H +4.04382005 −4.84844546 +1.05688687
H +2.54734049 −4.81961589 +2.05808029
H +2.69165597 −0.45348307 +0.54619010
H +3.41319880 −0.68129363 +2.12385288
H +1.65499147 +1.06091819 +2.26653549
H +1.15928084 −0.32479679 +3.24346854
H −1.00674905 +0.24499307 −0.36324566
H +0.73227109 +0.71732597 −0.40713563
19
* E = +4.222 kcal/mol ; (8) 317_293_034_180
C +0.00000000 +0.00000000 +0.00000000
C +0.00000000 +0.00000000 +1.50592360

C	+1.35052692	+0.00000000	+2.19732525
C	+2.42357611	−0.91783202	+1.63220544
C	+2.15462230	−2.39207034	+1.77960722
O	+0.84277208	−2.66124966	+1.68723414
C	+0.46650171	−4.02182207	+1.81462015
O	−1.02203888	+0.05390804	+2.14469938
O	+2.99247636	−3.23757135	+1.93558702
H	+0.79881038	−4.42969369	+2.78126070
H	+0.26919714	−1.00343460	−0.36828193
H	−0.62730183	−4.05300630	+1.74844754
H	+0.91453437	−4.62975629	+1.01346713
H	+2.58395033	−0.72643432	+0.55481273
H	+3.39828868	−0.73184478	+2.10815095
H	+1.71840590	+1.04237075	+2.14082260
H	+1.16341631	−0.20617869	+3.26352875
H	−1.00436834	+0.25147978	−0.36557423
H	+0.73925215	+0.70940776	−0.40794699

19

* E = +4.254 kcal/mol ; (9) 314_300_276_176

C	+0.00000000	+0.00000000	+0.00000000
C	+0.00000000	+0.00000000	+1.50247196
C	+1.35396499	+0.00000000	+2.19123452
C	+2.37271436	−0.99051604	+1.62806876
C	+1.85169364	−2.39681755	+1.73470623
O	+2.08504002	−2.91123358	+2.94646393
C	+1.55272273	−4.20289252	+3.18943958
O	−1.01968247	+0.00844506	+2.14901114
O	+1.24815734	−2.97902864	+0.87015376
H	+1.97514833	−4.94013930	+2.48946672
H	+0.26344259	−1.01316107	−0.34997413
H	+1.82053854	−4.46543124	+4.21969756
H	+0.45833631	−4.20436491	+3.07080128
H	+2.58539193	−0.78724033	+0.56710910
H	+3.31471090	−0.90596822	+2.19156641
H	+1.76352475	+1.02360206	+2.10327209
H	+1.17629694	−0.18575517	+3.26293342
H	−1.00140405	+0.25655068	−0.36993899
H	+0.75131337	+0.69474521	−0.41044647

19

* E = +4.440 kcal/mol ; (10) 314_071_047_180

C	+0.00000000	+0.00000000	+0.00000000
C	+0.00000000	+0.00000000	+1.50554805
C	+1.34910870	+0.00000000	+2.19793330
C	+2.37608386	−0.98055712	+1.64292595
C	+2.93800017	−0.65136292	+0.28641947
O	+3.30940191	+0.63664149	+0.20633676
C	+3.84496129	+1.04986767	−1.03854938
O	−1.02390330	+0.02440031	+2.14481848
O	+3.06358618	−1.42840297	−0.62180933
H	+3.10296166	+0.92346997	−1.84362362
H	+0.39567489	−0.95278043	−0.39128109
H	+4.10982732	+2.10880603	−0.93403894

H +4.73636595 +0.46010058 −1.30143726
H +3.23651202 −1.01727348 +2.33443920
H +1.97747649 −2.00582492 +1.58399132
H +1.75539369 +1.02613041 +2.12976927
H +1.15728241 −0.19805666 +3.26338837
H −1.02457293 +0.14068209 −0.36841048
H +0.65468078 +0.79705078 −0.39092746
19
* E = +5.658 kcal/mol ; (11) 315_183_319_181
C +0.00000000 +0.00000000 +0.00000000
C +0.00000000 +0.00000000 +1.50613457
C +1.34941413 +0.00000000 +2.19138394
C +2.36507836 −0.97127025 +1.60814420
C +3.67530660 −1.02330164 +2.34704988
O +4.08009766 +0.20081347 +2.71733289
C +5.32087027 +0.26486277 +3.39784420
O −1.02524937 +0.02176523 +2.14296221
O +4.30802243 −2.02089208 +2.56299209
H +6.13674069 −0.12057220 +2.76683318
H +0.30129490 −0.99238742 −0.37646523
H +5.49659998 +1.32061176 +3.63670847
H +5.29426938 −0.33230236 +4.32230062
H +1.98071170 −2.00349933 +1.57824423
H +2.60836881 −0.69704021 +0.56472690
H +1.75120326 +1.02814844 +2.12070728
H +1.17134640 −0.19346265 +3.26133916
H −1.00981780 +0.22296001 −0.36877599
H +0.72294772 +0.72767918 −0.40468247
19
* E = +6.969 kcal/mol ; (12) 147_293_180_358
C +0.00000000 +0.00000000 +0.00000000
C +0.00000000 +0.00000000 +1.49710589
C +1.36308584 +0.00000000 +2.16649748
C +1.33248454 +0.76932425 +3.46687369
C +1.10692262 +2.23808353 +3.19631839
O +1.05508594 +3.06519832 +4.25306051
C +1.15746601 +2.59370378 +5.57508724
O −1.01282192 +0.00054203 +2.15642353
O +0.97716250 +2.68566456 +2.08984616
H +2.13828395 +2.13069107 +5.77772627
H +0.72078888 −0.72925950 −0.40403903
H +1.04592905 +3.46557859 +6.23260064
H +0.36330157 +1.86910083 +5.82065011
H +0.50581687 +0.39473005 +4.09631822
H +2.25946911 +0.63284610 +4.05021587
H +1.62861850 −1.05729658 +2.35160282
H +2.12458190 +0.40462134 +1.48010548
H −1.00809616 −0.19383604 −0.38901234
H +0.33490765 +0.99935369 −0.32761420
19
* E = +7.455 kcal/mol ; (13) 174_294_141_346
C +0.00000000 +0.00000000 +0.00000000

C	+0.00000000	+0.00000000	+1.50031378
C	+1.35315064	+0.00000000	+2.17293414
C	+1.22448717	+0.13867755	+3.67443905
C	+0.65972767	+1.50346913	+4.01677839
O	−0.22860119	+1.61175997	+5.01318924
C	−0.88712932	+0.49389242	+5.56448620
O	−1.02266951	+0.02036169	+2.14832537
O	+1.03061067	+2.49126312	+3.44368096
H	−0.19925281	−0.15957483	+6.12861428
H	+0.72174999	−0.72649760	−0.40546789
H	−1.63635809	+0.88611027	+6.26482098
H	−1.40083586	−0.08979374	+4.78335834
H	+0.63200932	−0.69113777	+4.08251471
H	+2.22197153	+0.08702063	+4.14494669
H	+1.88437836	−0.92722902	+1.89051933
H	+1.95032141	+0.83077310	+1.75606965
H	−1.00870344	−0.20521248	−0.38159091
H	+0.31804204	+0.99590703	−0.35203665

19

* E = +8.464 kcal/mol ; (14) 177_179_180_000

C	+0.00000000	+0.00000000	+0.00000000
C	+0.00000000	+0.00000000	+1.50388057
C	+1.35815895	+0.00000000	+2.16107559
C	+1.28915940	+0.06150939	+3.66758881
C	+2.65491663	+0.04168847	+4.30313802
O	+2.70677096	+0.09523064	+5.64779522
C	+1.54748355	+0.16506363	+6.44210609
O	−1.02194276	+0.00426834	+2.15051334
O	+3.67694330	−0.01852359	+3.67877663
H	+0.89602699	−0.71513229	+6.30869479
H	+0.61753845	−0.82416273	−0.39209794
H	+1.88001025	+0.19374114	+7.48792605
H	+0.95711255	+1.07489910	+6.24039404
H	+0.73758690	+0.96268094	+3.99070546
H	+0.68056552	−0.77436059	+4.05662433
H	+1.91296497	−0.89762125	+1.83029786
H	+1.95722500	+0.83647160	+1.75641247
H	−1.02698529	−0.08671677	−0.37802036
H	+0.44980839	+0.93452229	−0.37464628

19

* E = +11.170 kcal/mol ; (15) 316_084_184_000

C	+0.00000000	+0.00000000	+0.00000000
C	+0.00000000	+0.00000000	+1.50318076
C	+1.34289204	+0.00000000	+2.21121044
C	+2.39689146	−0.94918062	+1.66400063
C	+3.18055850	−0.38801467	+0.49921961
O	+4.07596367	−1.20234640	−0.08670390
C	+4.29419851	−2.52610567	+0.33854729
O	−1.02433549	+0.02978497	+2.14312365
O	+3.05763356	+0.73349296	+0.09251510
H	+3.40249492	−3.16104157	+0.20051976
H	+0.43655330	−0.93423643	−0.39324037

H +5.10299999 −2.92924397 −0.28451031
H +4.61053695 −2.58000696 +1.39386680
H +3.12659831 −1.19127736 +2.45855227
H +1.95777674 −1.92568093 +1.38852701
H +1.73548753 +1.03190306 +2.15529596
H +1.13151592 −0.21396367 +3.26944574
H −1.03108557 +0.09251674 −0.36560191
H +0.63038929 +0.81704312 −0.38531031
19
* E = +11.269 kcal/mol ; (16) 033_061_170_359
C +0.00000000 +0.00000000 +0.00000000
C +0.00000000 +0.00000000 +1.50535873
C +1.34359875 +0.00000000 +2.21434254
C +2.49481656 +0.72670489 +1.54691350
C +2.20573026 +2.19791925 +1.36670686
O +3.21706770 +2.99174952 +0.97381541
C +4.51906707 +2.50504104 +0.75496436
O −1.01998161 −0.07059015 +2.14442005
O +1.11656705 +2.67078751 +1.53499406
H +4.54481511 +1.69771742 +0.00363808
H +0.72067099 −0.72955357 −0.40644516
H +5.11258066 +3.34693122 +0.37548000
H +4.98944960 +2.14304198 +1.68513488
H +3.41893637 +0.59123551 +2.13460139
H +2.72786409 +0.29954806 +0.55250929
H +1.15972744 +0.39461134 +3.22618520
H +1.61758628 −1.06425786 +2.34097458
H −1.01007754 −0.23256552 −0.36194113
H +0.28621432 +0.99699774 −0.37244251
19
* E = +12.701 kcal/mol ; (17) 045_060_090_004
C +0.00000000 +0.00000000 +0.00000000
C +0.00000000 +0.00000000 +1.50086685
C +1.35379848 +0.00000000 +2.19538795
C +2.39630561 +0.96664656 +1.63156407
C +1.90878258 +2.39742840 +1.69968499
O +2.17182865 +3.11369853 +2.80520293
C +2.94601155 +2.61745939 +3.87135864
O −1.01573840 −0.02441718 +2.15208670
O +1.25614648 +2.89329587 +0.82277140
H +3.99928069 +2.46356351 +3.58063947
H +0.75087974 −0.69643134 −0.40892854
H +2.91686739 +3.37779959 +4.66263893
H +2.54250943 +1.67740672 +4.28312965
H +3.35360240 +0.82343032 +2.15591032
H +2.58107069 +0.75342126 +0.56733016
H +1.15766155 +0.18341299 +3.26528158
H +1.74952689 −1.03017669 +2.11865098
H −1.00125207 −0.25799316 −0.36902426
H +0.26531148 +1.01348630 −0.34711518
19
* E = +13.037 kcal/mol ; (18) 064_036_069_351

C	+0.00000000	+0.00000000	+0.00000000
C	+0.00000000	+0.00000000	+1.50043713
C	+1.35602482	+0.00000000	+2.18095762
C	+2.15836298	+1.27745029	+1.89403222
C	+1.34215620	+2.55379871	+1.78394145
O	+0.76385167	+3.05496288	+2.88382034
C	+0.71387622	+2.37466167	+4.11769090
O	−1.01575300	+0.05332108	+2.15444111
O	+1.20912128	+3.12841520	+0.73786579
H	+1.69966336	+1.98622054	+4.42667316
H	+0.71027110	−0.73712549	−0.40751214
H	+0.38568739	+3.10550566	+4.86824276
H	−0.01858533	+1.55078357	+4.08185340
H	+2.94361716	+1.39568948	+2.66091759
H	+2.67315759	+1.19811860	+0.92484922
H	+1.18128418	−0.14633353	+3.25682292
H	+1.94458488	−0.86398334	+1.82567775
H	−1.01350461	−0.19042669	−0.37676556
H	+0.32623047	+0.99554060	−0.35114684

19

* E = +14.109 kcal/mol ; (19) 060_275_282_347

C	+0.00000000	+0.00000000	+0.00000000
C	+0.00000000	+0.00000000	+1.49986462
C	+1.35347257	+0.00000000	+2.19476130
C	+2.23599287	+1.21575874	+1.86503330
C	+3.03635207	+1.05177505	+0.59432795
O	+4.14264145	+0.28275722	+0.64953771
C	+4.71896698	−0.14741567	+1.86007388
O	−1.01702909	−0.00592462	+2.15060553
O	+2.71282271	+1.52683631	−0.45804887
H	+4.03255584	−0.75989974	+2.46867526
H	+0.66263921	−0.78856062	−0.39419494
H	+5.58537716	−0.76711250	+1.59407967
H	+5.07752883	+0.70064023	+2.46806817
H	+1.61490283	+2.11629691	+1.73716695
H	+2.90457431	+1.41451715	+2.71755833
H	+1.14442053	−0.03657941	+3.27469112
H	+1.87576891	−0.93832796	+1.92679508
H	−1.02385152	−0.14121397	−0.37024374
H	+0.41100927	+0.94856021	−0.38489557
